# Management of Retained Epidural Catheter Fragments: A Narrative Review of Individual Patient Data

**DOI:** 10.3390/jcm14124265

**Published:** 2025-06-16

**Authors:** Felix Corr, Yasser F. Almealawy, Silvio Heinig, Linda Bättig, Erik Schulz, Nader Hejrati, Lorenzo Bertulli, Stephan Heisinger, Oliver Bozinov, Martin N. Stienen, Stefan Motov

**Affiliations:** 1 Department of Neurosurgery & Interdisciplinary Spine Center, HOCH Health Ostschweiz, Cantonal Hospital St. Gallen, Rorschacher Strasse 95, 9007 St. Gallen, Switzerland; 2 Faculty of Medicine, University of Kufa, Kufa P.O. Box 21, Iraq; 3 Department of Orthopedics and Trauma Surgery, Medical University of Vienna, 1090 Vienna, Austria

**Keywords:** epidural catheter, epidural anesthesia, spinal anesthesia, breakage, fragment

## Abstract

**Background/Objectives:** Retained epidural catheter fragments are an infrequent but clinically relevant complication of neuraxial anesthesia. Optimal management remains undefined, with limited evidence guiding treatment selection or risk stratification. This systematic review synthesized individual patient data to compare treatment strategies, examine surgical outcomes, and determine predictors of intervention. **Methods:** A systematic review was conducted across six databases in accordance with PRISMA guidelines (PROSPERO: CRD420025638305). Adult cases of retained epidural catheter fragments were included. Functional outcomes were standardized using modified MacNab, McCormick, and Therapy–Disability–Neurology (TDN) scores. Predictors of surgery and detectability were assessed using univariate and multivariate logistic regression models with Firth correction. **Results:** Forty studies comprising 51 patients were included. Conservative management was chosen in 23 cases (45%); 39.1% (*n* = 9) ultimately required delayed surgery due to symptom onset during follow-up. Surgical removal (*n* = 28, 55%) was safe and yielded excellent outcomes in 95.8% of cases. Fragment length was significantly associated with increased odds of surgery (OR = 1.05, 95% CI: 1.01–1.10, *p* = 0.04), while catheter material was associated with surgery in univariate analysis (OR = 2.49, 95% CI: 1.08–9.00, *p* = 0.03). An MRI demonstrated the highest diagnostic accuracy (AUC = 0.859, cutoff = 70 mm catheter length), outperforming CT (AUC = 0.611) and X-ray (AUC = 0.533). Across all patients, 84.3% achieved “Excellent” recovery per MacNab, with no neurological deterioration in any surgical case. **Conclusions:** Surgical removal of retained epidural catheter fragments is safe and effective in symptomatic patients. Conservative management is viable for asymptomatic cases under structured surveillance. Catheter material and fragment length may dictate imaging selection and treatment decisions.

## 1. Introduction

Epidural catheterization is widely used for perioperative neuraxial analgesia, offering effective pain control while reducing opioid-related morbidity [1]. However, catheter fragmentation during removal is a rare but clinically relevant complication, with an estimated incidence of 0.002–0.04% [2,3].

While many retained fragments remain asymptomatic, cases of progressive neurological deterioration and inflammatory reactions have been reported [4,5]. Therefore, optimal management of retained epidural catheter fragments remains unclear [6]. Although conservative treatment with observation is frequently advocated for in asymptomatic cases, the risk of late-onset neurological symptoms or catheter migration raises concerns about long-term surveillance [4,7].

Surgical removal is generally indicated for symptomatic cases, yet no standardized criteria exist for patient selection, risk stratification, or the preferred retrieval technique. Importantly, there is a lack of consensus among clinicians and no formal clinical guidelines to inform decision-making in these cases. The absence of high-quality longitudinal data has limited the development of evidence-based recommendations.

The aim of this systematic review was to compare conservative and surgical management strategies for retained epidural catheter fragments, evaluate outcomes across surgical techniques, assess the diagnostic performance of imaging modalities, and identify factors influencing the need for surgical intervention.

## 2. Materials and Methods

This systematic review was conducted in accordance with the Cochrane Handbook for Systematic Reviews of Interventions and is reported following the Preferred Reporting Items for Systematic Reviews and Meta-Analyses (PRISMA) guidelines (Table S1) [8,9]. The protocol was prospectively registered in PROSPERO (CRD420025638305) [10].

### 2.1. Eligibility Criteria

This review included studies investigating adult patients (≥18 years) with torn epidural catheters managed conservatively or surgically. Study selection followed the Population, Intervention, Comparison, Outcomes, and Study (PICOS) framework (Table S2) [11]. Eligible designs comprised randomized and non-randomized controlled trials, cohort studies, longitudinal studies, cross-sectional studies, and case reports. Primary outcomes included catheter removal success rates, patient safety, and functional outcomes such as neurological deficits.

Inclusion criteria for full review were as follows: (1) studies on adult patients (≥18 years) undergoing surgical or conservative management (e.g., observation) for retained epidural catheter fragments, (2) studies reporting success rates and complications, and (3) publications in English. Studies were excluded if they involved pediatric populations, non-English publications, lacked sufficient data on removal techniques or outcomes, or described catheter kinking or knotting without actual breakage and retention (Table S3).

### 2.2. Information Sources and Search Strategy

A systematic, multi-database literature search was performed across PubMed, Google Scholar, Cochrane Library, ScienceDirect, Web of Science, and Bielefeld Academic Search Engine (BASE), with additional manual screening of relevant articles. Bibliographies of included studies and gray literature, including conference abstracts and preprints, were reviewed for supplementary sources. To mitigate selection bias and ensure completeness, forward and backward citation tracking (snowball searching) was conducted. The final search was completed on 15 March 2025.

A structured search strategy was developed using Boolean operators (AND, OR) to systematically link terms. Controlled vocabulary (e.g., MeSH terms, Emtree) and free-text keywords were combined to optimize sensitivity and specificity, with truncation and wildcards (*) employed to capture variations. Searches were restricted to titles, abstracts, and keywords where database functionality allowed (Table S4). The strategy was peer-reviewed (S.M.) using the Peer Review of Electronic Search Strategies (PRESS) checklist [12]. No language, date, or publication status filters were applied to reduce the selection bias. Search results were deduplicated using EndNote 20 (Clarivate Analytics, Philadelphia, PA, USA) and screened following pre-specified eligibility criteria.

### 2.3. Selection Process and Data Collection Process

The initial search identified 448 articles, which were screened independently by two reviewers (F.C. and Y.A.) using the PICOS framework to determine eligibility. Titles and abstracts were assessed for relevance, and publications were excluded if they addressed non-epidural catheter interventions, were not in English language, or consisted of narrative reviews or case descriptions lacking primary data. Full-text articles were then retrieved and evaluated against predefined eligibility criteria (Table S3), with inclusion and exclusion decisions documented systematically. A pilot screening phase was conducted on 50 randomly selected abstracts to ensure inter-reviewer agreement before formal screening. Inter-rater reliability for study inclusion was assessed using Cohen’s kappa coefficient. Discrepancies at any stage were resolved through consensus discussions or, when necessary, adjudication by a third reviewer (S.M.) No automation tools or artificial intelligence-assisted screening methods were utilized to mitigate the risk of algorithmic bias or inadvertent exclusion of relevant studies.

Extracted data included article details (author, title, year, DOI, study design), patient baseline characteristics (age, gender, comorbidities), catheter-related factors (material, indication for placement, spinal level of insertion, probable cause of fragmentation), imaging modalities used, surgical details (type of surgery), reported complications, relevant clinical scores (see Section 2.5), and follow-up outcomes.

Surgical interventions were classified into three distinct categories: open surgery (full laminectomy or wide exposure with bony decompression), endoscopic retrieval (minimally invasive, camera-guided approaches), and limited open dissection. The latter term refers to procedures involving soft-tissue exposure through skin, fascia, and paraspinal muscle layers without any bone work (e.g., no laminectomy, laminotomy, or decompression), performed to localize and extract the catheter fragment.

For all included studies, individual patient data were reconstructed from clinical narratives and tabular data presented in published case reports and small case series. Only explicitly reported patient-level variables were extracted; no imputations or extrapolations were made beyond the information available in the source publications.

### 2.4. Certainty, Study Risk of Bias Assessment, and Reporting

The Joanna Briggs Institute (JBI) Critical Appraisal Checklist for Case Reports was used to assess study quality systematically [13]. Two independent reviewers (F.C. and Y.A.) systematically evaluated each study using a standardized assessment form, recording domain-specific judgments and an overall appraisal score (Table S5). Discrepancies were resolved through structured discussions, and if consensus was not reached, a third reviewer (S.M.) adjudicated.

### 2.5. Outcome Parameters

Due to the absence of standardized, quantifiable outcome parameters in the included studies, validated clinical scoring systems were applied to ensure a systematic assessment of therapy-related complications, neurological impairment, and post-treatment recovery. The Therapy–Disability–Neurology (TDN) score was used to classify therapy-related complications, neurological status, and disability severity following epidural catheter insertion, ranging from Grade 1 (minor complications without lasting disability) to Grade 4 (severe neurological impairment with permanent functional loss) [14]. The McCormick Scale provided a structured functional classification of neurological impairment, particularly in spinal cord pathology, categorizing patients based on mobility and sensory deficits, with Grade I representing full independence and Grade IV indicating severe disability requiring assistance [15]. The modified MacNab Criteria were applied to evaluate post-treatment functional recovery, stratifying outcomes into Excellent (complete pain relief, unrestricted activity), Good (mild symptoms not interfering with function), Fair (persistent symptoms limiting function), and Poor (no improvement, persistent pain with functional impairment) [16].

In addition to individual outcome grading, a structured synthesis of clinical recommendations reported across included studies was performed to develop practical guidance. Consensus synthesis was conducted by extracting explicit management recommendations from included case reports and qualitatively grouping them by clinical domain. No formal Delphi or structured survey methodology was used. Levels of agreement were defined as follows: strong consensus (≥75% concordance), moderate consensus (50–74%), and limited consensus or evidence (<50% agreement or inconsistent reporting). Supporting and opposing viewpoints were documented, and domains with unresolved or conflicting evidence were annotated accordingly

### 2.6. Data Analysis

Data were collected and managed using Excel Version 16.01 (Microsoft, Redmond, WA, USA). Data preparation, statistical analysis, and visualization were conducted using R software (version 4.0.4, R Foundation for Statistical Computing, Vienna, Austria) and GraphPad Prism (Version 10.0.3, GraphPad Software, Inc., San Diego, CA, USA).

Descriptive statistics are presented as means with standard deviations (SD) for continuous variables and as absolute numbers with corresponding proportions (%) for categorical variables. Outcome data are reported with 95% confidence intervals (95% CI) where applicable. The normality of continuous data was assessed using a Shapiro–Wilk test. Unpaired categorical and binary variables were analyzed using a Fisher’s exact test in contingency tables. Continuous variables were compared between the two groups using an unpaired *t*-test, while a one-way analysis of variance (ANOVA) was employed to assess differences among surgical subgroups.

The sensitivity for each imaging modality was calculated as the proportion of correctly identified cases per patient to evaluate diagnostic accuracy. To evaluate the predictive value of fragment length for detectability across imaging modalities, receiver-operating characteristic (ROC) curves were constructed for each modality. Optimal fragment length cut-off values were identified using the Youden Index. To assess predictors of fragment detectability, univariate and multivariate logistic regression models were fitted with fragment length and catheter material as independent variables. Firth’s penalized likelihood correction was applied to reduce small-sample bias.

Finally, univariate and multivariate logistic regression models were utilized to assess age, gender, comorbidities, catheter material, fragment length, and the spinal level as independent predictors of both primary surgical intervention and secondary surgery following initial conservative management. All statistical tests were two-tailed, with a significance threshold set at *p* < 0.05.

### 2.7. Sensitivity Analyses

To assess model robustness in the setting of small-sample bias and potential outlier influence, a leave-one-out sensitivity analysis was performed on the multivariate logistic regression model evaluating predictors of surgical intervention.

To account for potential small-sample bias and separation issues, Firth’s penalized likelihood logistic regression was additionally performed as a sensitivity analysis for models assessing predictors of both primary surgical intervention and secondary surgery following initial conservative management.

## 3. Results

### 3.1. Study Characteristics

**Figure 1:**
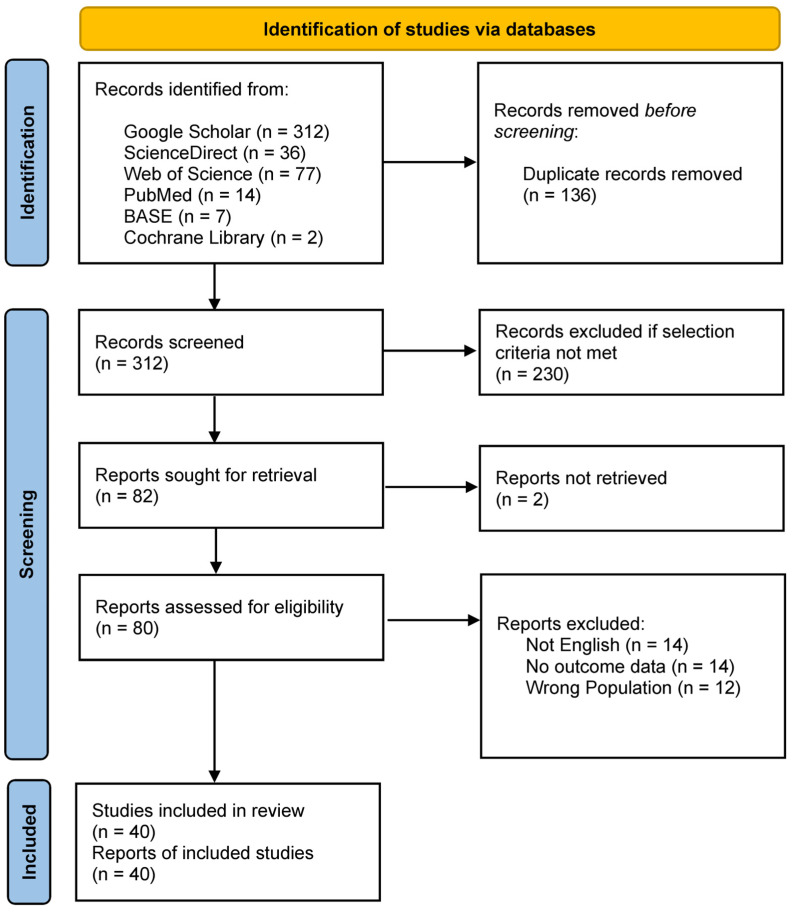
PRISMA flow chart for the systematic review detailing the database searches, the number of records screened, and the studies included.

A total of 448 records were identified through systematic database searches. After removing 136 duplicates, 312 records remained for title and abstract screening. Of these, 230 were excluded for not meeting the eligibility criteria. The full texts of the remaining 80 reports were assessed for eligibility, resulting in the inclusion of 40 studies in the final systematic review. The flow chart of data selection is shown in Figure 1.

A total of 51 individual patients with retained epidural catheter fragments were included, comprising 23 patients (45.1%), who were managed conservatively [4,5,7,17,18,19,20,21,22,23,24,25,26,27,28,29,30,31,32] and 28 patients (54.9%), who underwent surgical removal [2,33,34,35,36,37,38,39,40,41,42,43,44,45,46,47,48,49,50,51,52]. An overview of included studies is provided in Table S6, and pooled baseline patient characteristics are summarized in Table 1.

The mean age was similar between the conservative and surgical management groups (45.3 ± 23.8 vs. 45.4 ± 17.9 years, respectively; *p* = 0.99), and did not differ in the overall cohort (45.3 ± 21.0 years). There tended to be more female patients in the conservative than in the surgical cohort (78.3% vs. 53.6%, *p* = 0.08). Among available data, cardiovascular (13.7%), musculoskeletal (13.7%), and oncologic (9.8%) conditions were most commonly reported.

Clinical indications for epidural catheterization included analgesia during delivery (39.2%), orthopedic surgery (35.3%), and gastrointestinal surgery (15.7%), with comparable distributions across treatment groups. Regarding catheter characteristics, polyamide (nylon) catheters tended to be more often utilized in the conservative group (52.2% vs. 14.3%, *p* = 0.06). Retained fragment length differed significantly between groups, with longer fragments observed in the surgical cohort (103.3 ± 33.1 mm vs. 66.3 ± 49.7 mm; *p* = 0.02). Spinal level data indicated that most fragments were retained in the mid-lumbar levels, L3/L4 (31.4%) and L2/L3 (21.6%), although thoracic placements were also included (total 19.6%, see Table 1).

Functional outcomes were generally favorable with 84.3% of all patients, based on the modified MacNab Criteria achieving excellent outcomes, including 87.0% in the conservative group and 95.8% in the surgical group (OR = 2.55; 95% CI: 0.30–54.21; *p* = 0.44). Similarly, according to the McCormick Scale, 91.3% of conservatively treated patients and 95.8% of surgical patients were functionally independent (Grade I; *p* = 0.34). None of the included studies reported any medico-legal claims or litigation related to the cases described.

### 3.2. Quality Assessment

Most studies provided comprehensive patient demographics, detailed clinical descriptions, and clearly documented interventions and outcomes (Table S5). However, significant heterogeneity was observed in the completeness of follow-up, reporting of adverse events, and consideration of differential diagnoses. Overall evidence quality was limited; case reports were classified as Level 4 according to the Oxford Centre for Evidence-Based Medicine (OCEBM). One case series met the criteria for Level 4 evidence [48], and one retrospective cohort study was categorized as Level 3 [28]. Methodological rigor varied widely, with some studies demonstrating robust clinical reasoning and clearly defined outcomes, while others lacked critical information regarding intervention protocols, long-term follow-up, and standardized outcome assessments.

### 3.3. Characteristics of Catheter Fragmentation

In total, 43 instances of catheter fragmentation were described. The most prevalent was mechanical stress during removal, accounting for 17 cases (39.5%), and included removal against resistance (*n* = 5, 11.6%), withdrawal through the Tuohy needle (*n* = 5, 11.6%), simultaneous extraction of the catheter and needle (*n* = 3, 7.0%), and excessive traction (*n* = 4, 9.3%). Technical errors during insertion or handling were identified in ten cases (23.3%), involving multiple insertion attempts or redirections (*n* = 3, 7.0%), excessive looping or manipulation (*n* = 5, 11.6%), and improper handling during placement (*n* = 2, 4.7%).

**Figure 2:**
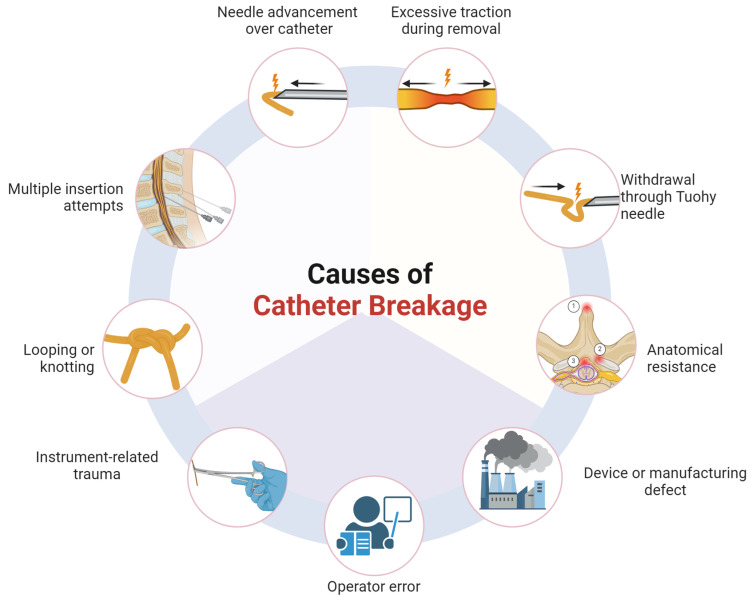
Illustration of mechanistic pathways of epidural catheter fragmentation. Anatomical resistance was reported as a cause of catheter breakage. Key sites included the following: (1) spinous processes (e.g., Baastrup disease), (2) facet joints, and (3) ligamentum flavum or dura (created with BioRender.com).

Instrument-related trauma contributed to six cases (14.0%), primarily due to shearing with forceps or clamps (*n* = 3, 7.0%), suspected transection by the Tuohy needle (*n* = 2, 4.7%), and microdamage from gripping instruments (*n* = 1, 2.3%). Anatomical or pathological resistance was cited in six cases (14.0%), including kinking along the dura or in the presence of adhesions (*n* = 2, 4.7%) and structural entrapment, such as coiling within the facet joint or ligamentous entanglement (*n* = 4, 9.3%). Operator-related factors, such as procedural mishandling or involvement of untrained personnel, were documented in four cases (9.3%). The overall mechanistic pathways are summarized visually in Figure 2.

### 3.4. Imaging Modalities

The imaging modality performances varied, with overall detection rates of 34.6% for X-ray, 56.5% for CT, and 71.4% for MRI.

X-ray detectability was primarily influenced by the catheter material, while the fragment length showed no significant association. Although the overall model did not reach statistical significance (*p* = 0.17), the nylon catheters were significantly less likely to be detected compared to the polyurethane catheters (OR = 0.06, 95% CI: 0.0004–0.81, *p* = 0.033). The fragment length was not predictive of X-ray visibility (OR = 1.01 per mm, 95% CI: 0.98–1.06, *p* = 0.48), and other materials did not demonstrate statistically significant differences.

The CT detectability was significantly influenced by both the fragment length and catheter material (*p* = 0.037). Longer fragments were more likely to be visualized (OR = 1.04 per mm, 95% CI: 1.00–1.26, *p* = 0.041). Compared to polyurethane catheters, the polyamide (nylon) catheters were markedly less detectable (OR = 0.014, 95% CI: 0.00003–0.41, *p* = 0.009).

The MRI detectability was not significantly associated with the fragment length or catheter material (*p* = 0.18), likely reflecting the small sample size (*n* = 8). However, the fragment length showed a borderline association with increased detectability (OR = 1.06 per mm, 95% CI: 1.00–1.38, *p* = 0.067)

**Figure 3:**
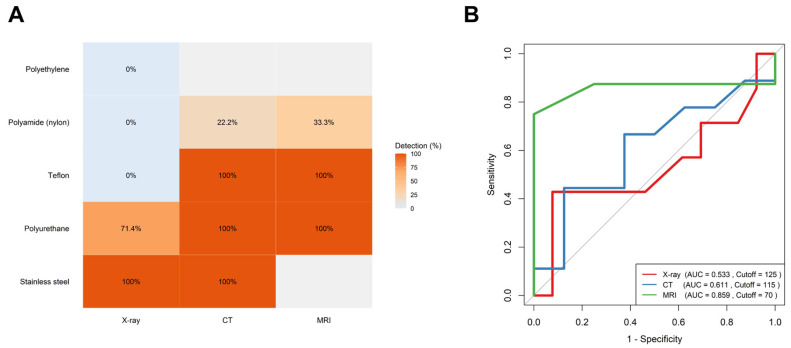
Comparative detection of retained epidural catheter fragments across imaging modalities: (**A**) Heatmap showing detection rates (%) of various catheter materials across imaging modalities. MRI and CT achieved full detection for Teflon, polyurethane, and stainless-steel fragments, while polyamide and polyethylene showed poor visibility, particularly on X-ray. (**B**) Receiver-operating characteristic (ROC) curves comparing diagnostic performance. MRI (green) demonstrated the highest diagnostic accuracy (AUC = 0.859, cutoff = 70 mm), outperforming CT (blue, AUC = 0.611) and X-ray (red, AUC = 0.533).

A comparison of the detection rates across materials revealed significant differences for X-ray (*p* = 0.0038) and CT (*p* = 0.0182) but not for MRI (*p* = 0.4286). Specifically, the polyurethane catheters demonstrated superior detectability (X-ray 71.4% vs. CT and MRI 100%) compared to the polyamide (X-ray 0%, CT 22.2%, and MRI 33.3%) and polyethylene ones, which exhibited poor radiographic visibility across modalities (Figure 3A). The MRI showed the highest discriminative performance based on the ROC analysis (AUC = 0.859, optimal cutoff = 70 mm), followed by the CT (AUC = 0.611, cutoff = 115 mm), while the X-ray demonstrated a poor diagnostic utility (AUC = 0.533, cutoff = 125 mm) (Figure 3B).

### 3.5. Conservative Management

**Figure 4:**
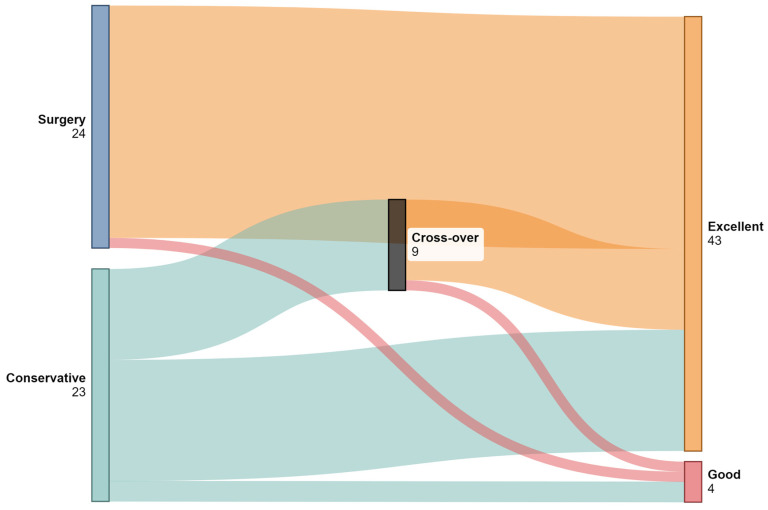
Sankey diagram illustrating patient flow between conservative management, surgical intervention, and cross-over (initially conservative patients who later underwent surgery). Final outcomes are classified using modified MacNab Criteria into “Excellent” and “Good” categories. Only patients with complete outcome data were included in the visualization. (Created with SankeyMatic).

Of the 23 patients managed conservatively following epidural catheter fracture, 14 (60.9%) were successfully observed without further intervention [17,18,20,21,22,23,25,27,28,30], while 9 (39.1%) subsequently required surgical removal [4,5,7,19,24,26,29,31,32]. The mean duration of follow-up in the conservative group was 912.0 days (range: 60–3960 days; SD: 1275), compared to 152.2 days (range: 4–360 days; SD: 190.0) in the surgical group (*p* = 0.136). The distributions of initial management strategies (conservative vs. surgical), cross-over to surgery, and functional outcomes based on the modified MacNab Criteria are illustrated in Figure 4.

Baseline demographics did not differ significantly between groups, with a mean age of 46.0 ± 2.2 years in the observation group and 43.6 ± 21.3 years in the cross-over group (*p* = 0.78), in addition to a comparable proportion of females (*n* = 11, 78.6% vs. *n* = 7, 77.8%, *p* > 0.99). Polyamide (nylon) catheters were markedly more common among patients who remained under observation (*n* = 11, 78.6%) compared to those who underwent secondary surgery (*n* = 1, 11.1%; *p* = 0.002). Additionally, the mean retained fragment length was significantly greater in the surgical group (96.4 ± 60.5 mm vs. 50.0 ± 35.5 mm; *p* = 0.04). A detailed overview of clinical and procedural characteristics in both cross-over and conservative patients is presented in Table 2.

Among the nine patients requiring secondary surgery, the symptom onset ranged from 9 days to 18 years post-catheter fracture (9 days [5], 7 weeks [7], 7 months [19], 18 months [4], 8 years [32], and 18 years [29]). Notable findings were low lumbar pain [24,26], with femoral nerve sensory loss [19], and spinous process tenderness exacerbated by spinal flexion [26]. One patient developed bilateral lower limb paresis after 18 years [29].

### 3.6. Surgical Management

Among 37 patients undergoing surgical removal of a retained epidural catheter fragment, three primary techniques were employed: open surgery (*n* = 13, 35.1%) [4,7,19,29,34,38,42,45,51,52], endoscopic retrieval (*n* = 12, 32.4%) [24,31,35,47,48,50], and limited open dissection (*n* = 12, 32.4%) [2,5,26,33,36,37,39,40,43,44,49]. Baseline characteristics, including age (mean 36.2–50.8 years, *p* = 0.27) and sex distribution (female: 58.3–61.5%, *p* > 0.99), were comparable across groups. Surgical indications included patient preference in ten cases (27.0%) [24,26,35,36,38,40,44,49,50,51], persistent or progressive back pain in seven patients (18.9%), physician recommendation based on clinical judgment in six cases (16.2%) [34,39,40,42,44,45], and evidence of catheter migration on follow-up imaging in one patient (2.7%) [7]. The retained fragment length was numerically longest in the limited open dissection group (112.7 mm ± 39.5 mm) and shortest in the endoscopic group (66.7 mm ± 25.2 mm, *p* = 0.24). Functional outcomes were uniformly favorable.

Open surgery, endoscopic retrieval, and limited open dissection were each associated with favorable outcomes. Excellent recovery was achieved in 84.6% of patients who underwent open surgery and in all of those treated with endoscopic retrieval or limited open dissection. Functional independence (McCormick Grade I) was observed in all patients with available postoperative data. Importantly, no postoperative neurological deterioration occurred in any surgical subgroup. A full comparative overview of surgical subgroups is provided in Table 3**.**

In univariate analysis, catheter material (OR = 2.49, 95% CI: 1.08–9.00, *p* = 0.03) and fragment length (OR = 1.03 per mm, 95% CI: 1.01–1.06, *p* = 0.0006) were significantly associated with surgical intervention. In multivariate analysis, only fragment length remained independently associated with surgery (OR = 1.05 per mm, 95% CI: 1.01–1.10, *p* = 0.04), while catheter material did not retain statistical significance (*p* = 0.33). Full model results are presented in Table 4.

In the leave-one-out sensitivity analysis, most covariates yielded stable estimates across iterations. However, polyurethane catheter material (range: −3.26 to 4.19; SD 1.24) and fragment length (range: −2.77 to 0.04; SD 0.42) showed considerable variability, suggesting susceptibility to individual data points (Table S7). Estimates for age, gender, and spinal level remained robust. When applying Firth’s penalized logistic regression, confidence intervals widened—as expected with this conservative approach—but the direction and magnitude of key associations largely mirrored those of the standard model (Table S8).

### 3.7. Management Consensus

To inform clinical decision-making, a structured synthesis of author recommendations from individual case reports was performed. This qualitative matrix approach allowed for consolidation of expert opinion into actionable guidance. A strong consensus emerged in favor of comprehensive documentation and transparent communication with patients and providers, supported by routine follow-up to mitigate long-term medico-legal and clinical risks. The MRI was endorsed as one of the primary imaging modalities for fragment localization. Conservative management was deemed acceptable in asymptomatic patients, contingent on informed consent and structured surveillance, whereas surgical intervention was recommended in the presence of neurological symptoms, persistent cerebrospinal fluid leakage, infection risk, intrathecal positioning, cutaneous extrusion, or concurrent anticoagulation, yielding moderate consensus. The timing of surgery remained variably addressed. Early intervention (within days to weeks) was often advised to prevent fibrosis and adhesion, though not universally endorsed. Follow-up recommendations showed limited evidence and consensus, with annual review suggested in low-risk cases and individualized intervals based on symptom evolution or procedural risk. The complete matrix of consensus domains, agreement levels, and supporting sources is provided in Table 5.

### 3.8. Illustrative Case

**Figure 5:**
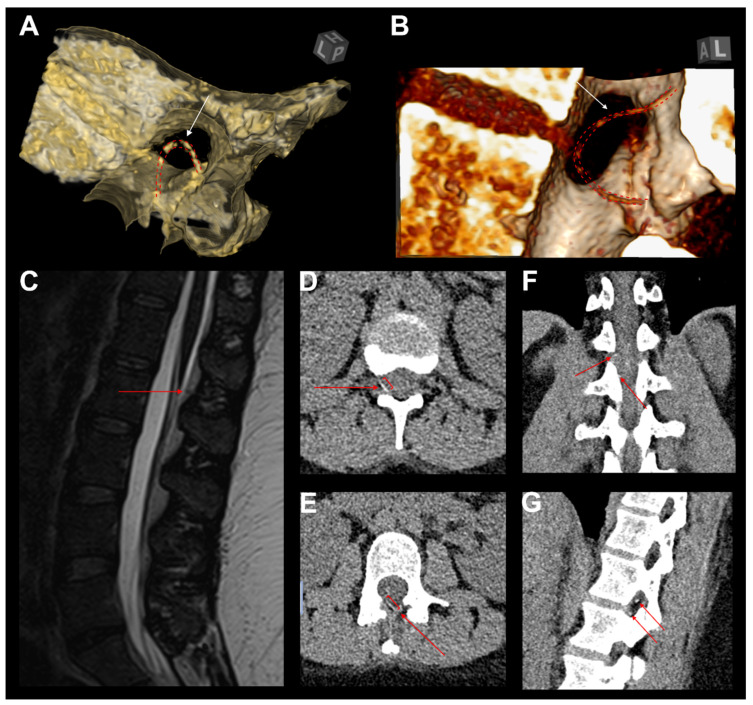
Illustrative case of catheter looping and consecutive breakage. (**A**,**B**) Three-dimensional CT reconstructions demonstrating a retained epidural catheter fragment (white arrows and red dashed line) with looping and entrapment at the interlaminar space, contributing to failed removal and subsequent breakage. (**C**) Sagittal T2-weighted MRI revealing the retained catheter fragment (red arrow) within the posterior epidural space, without evidence of significant spinal cord compression. (**D**,**E**) Axial CT images illustrating the fragmented catheter segment (red arrows) embedded in the epidural space, extending into the right neuroforaminal area. (**F**,**G**) Coronal and sagittal CT reconstructions confirming the catheter’s intralaminar positioning (red arrows), demonstrating intersegmental looping. The catheter follows a bi-segmental aberrant trajectory, which likely contributed to retrieval resistance and mechanical failure.

A 26-year-old woman (body-mass index: 41.8) presented with a retained epidural catheter fragment following labor analgesia, incidentally discovered after inadvertent catheter disruption during removal. Cross-sectional imaging via CT and MRI confirmed a 90 mm residual fragment, localized in the epidural space spanning T12 to L2, with no evidence of hematoma, intrathecal migration, or neural compression (Figure 5).

Although the patient remained neurologically intact, with a normal gait, full range of spinal motion, and preserved strength and sensation, she reported persistent localized thoracolumbar discomfort, worsened by movement and deep inspiration. Additionally, she expressed significant psychological distress related to the retained foreign body, despite reassurance and counseling. In the absence of objective neurological deficits, conservative management was initially recommended.

However, due to the patient’s insistence, motivated by both somatic symptoms and psychological burden, surgical removal was pursued. A unilateral biportal endoscopic approach (UBE) was performed at the L1/2 level, achieving successful identification and extraction of the retained catheter fragment. The decision to utilize the UBE technique was guided by its ability to systematically explore distinct anatomical planes, which was particularly advantageous given the fragment’s mediolateral orientation at the thoracolumbar junction. Uncertainty regarding whether the catheter fragment lay epidurally or external to the ligamentum flavum further supported the selection of this approach. The patient had an uneventful postoperative recovery, was discharged on the first postoperative day, and remained asymptomatic at follow-up [50].

### 3.9. Recommendations

Management of retained epidural catheter fragments should be primarily guided by clinical symptomatology and patient preference rather than fragment length alone. Although longer fragments were associated with surgical intervention in univariate analysis, this relationship did not persist in multivariate models, underscoring the limited discriminatory value of length in isolation. Fragment location, configuration, and material composition likely exert greater clinical influence, though these variables were not systematically evaluated. Shorter fragments may elicit significant symptoms, while longer fragments can remain asymptomatic, highlighting the limitations of size-based thresholds and the importance of individualized clinical assessment.

Comprehensive documentation and clear communication with both patients and healthcare providers are essential for ensuring continuity of care and medicolegal safety. MRI is recommended as the primary imaging modality for fragment localization, with CT reserved for cases where MRI is inconclusive or contraindicated.

Shared decision-making is critical. Patients should be counseled on the potential natural history of retained fragments, including delayed complications such as fibrosis, granuloma formation, and nerve irritation, which may emerge over variable latency periods. Management strategies must remain dynamic, with evolving symptoms or psychological distress prompting re-evaluation.

Surgical removal is indicated in the presence of neurological deficits, persistent cerebrospinal fluid leakage, intrathecal fragment migration, infection risk, external protrusion, or anticipated need for anticoagulation therapy. Early intervention is recommended to minimize the risk of fibrosis and adhesions. Surgical techniques should be tailored to anatomical considerations, catheter characteristics, and surgeon expertise, with minimally invasive endoscopic approaches favored when feasible due to their efficacy and soft-tissue preservation.

**Figure 6:**
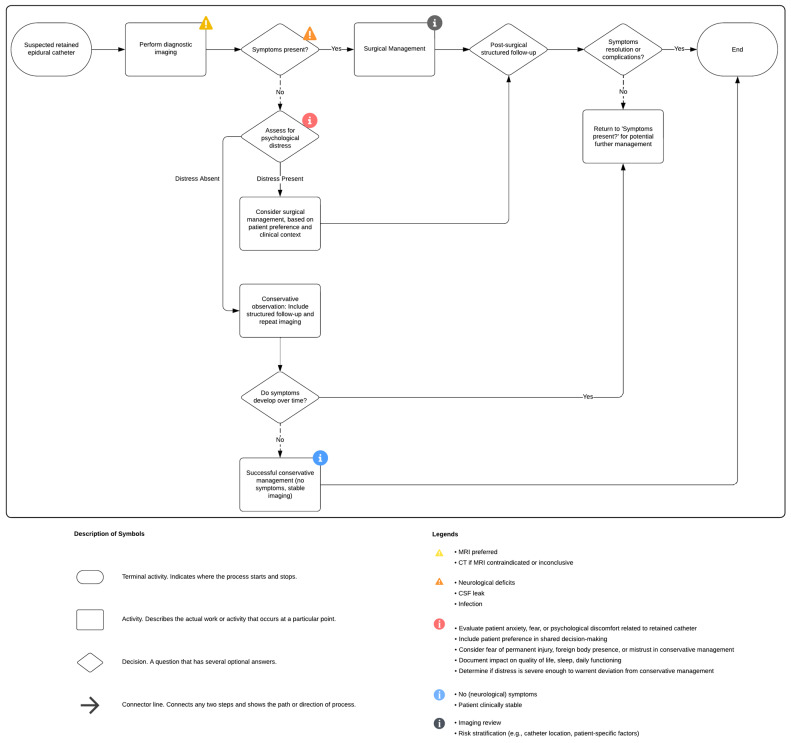
Flow chart of clinical management of broken epidural catheters (created with Lucidchart).

In asymptomatic patients, conservative management within a structured surveillance protocol remains appropriate. The follow-up should include periodic clinical evaluation and imaging, with annual intervals recommended as a baseline and shorter intervals based on individual risk. Clear criteria for re-evaluation, such as few symptoms, unresolved anxiety, or a change in patient preference, should be established. Figure 6 illustrates a structured clinical flowchart for the management of retained epidural catheter fragments.

## 4. Discussion

This systematic review offers a detailed patient-level synthesis of the current literature on retained epidural catheter fragments, a rare but potentially consequential complication of neuraxial anesthesia. Drawing on 51 individual patient cases, our review critically evaluates the clinical rationale underpinning both conservative and surgical approaches, compares functional outcomes across surgical techniques, and identifies procedural and patient-level risk factors associated with treatment escalation. Despite inherent limitations in the existing literature, the present analysis yields several clinically relevant and actionable insights.

### 4.1. Key Findings

Our review suggests that surgical management, whether open, endoscopic, or through limited dissection, is effective and generally safe in selected patients, with no postoperative neurological decline and frequent symptom resolution observed in this cohort. These findings support surgery as a viable option when clinically indicated.

However, this observation should be interpreted with caution, as it may be subject to reporting bias, as unfavorable or complicated cases are less frequently reported in the literature. Conservative management, supported by structured follow-up, appears to be a reasonable initial strategy. However, the need for delayed surgery in 39 percent of conservatively treated patients highlights that symptom progression may occur in a significant subset, reinforcing the importance of close monitoring and timely reassessment. The longer mean follow-up in the conservative group (912 vs. 152 days) suggests that late surgical conversions were unlikely to have been missed, supporting the robustness of the observed conservative success rate.

Both the fragment length and catheter material were associated with surgical intervention in univariate analysis; however, only the fragment length remained significant in multivariate logistic regression. The link between the fragment length and surgical intervention likely reflects selection bias, serving more as a proxy for clinical concern than a true independent risk factor. To evaluate the robustness of these findings, the Firth logistic regression was performed as a sensitivity analysis. In this model, the polyurethane catheter material, but not the fragment length, was significantly associated with surgery, suggesting model-dependent variability. The sensitivity analysis highlighted the variability in predictor stability of the fragment length, while the polyurethane material showed a more consistent association (Tables S7 and S8). Nylon catheters, overrepresented in conservatively managed cases, may provoke less tissue response. Prior research has shown polyurethane to be significantly more elastic than nylon, which may improve mechanical resilience, but may complicate retrieval once fragmented [53].

Although epidural catheter materials are generally regarded as biocompatible [51], emerging reports challenge this assumption. Instances of subcutaneous effusion [5], granuloma formation [4], reactive fibrosis [32], and dural adhesions [7] suggest that retained fragments can provoke localized inflammation in some patients, potentially complicating delayed removal. These inflammatory changes may evolve gradually and could potentially increase the technical complexity of delayed surgical removal. Fibrotic encapsulation and tissue ingrowth may obscure fragment location and pose challenges to safe retrieval, though evidence remains limited [4].

The MRI showed the highest diagnostic accuracy for fragment detection (AUC 0.859; threshold 70 mm), outperforming CT and X-ray. However, these findings are based on a small sample and may be influenced by variability in scanner type, imaging protocols, or reader expertise. While limited, the available data support MRI as the preferred modality, with CT reserved for specific cases, such as metal fragments or inconclusive MRI. In addition, MRI is particularly advantageous in vulnerable populations, such as post-labor nursing mothers, pregnant women, and young patients, due to its lack of ionizing radiation.

In select cases, surgery was performed at the patient’s request due to psychological distress, despite the absence of neurological symptoms [35,36,49,51]. While patient well-being and autonomy are central to shared decision-making, psychological burden alone should not justify invasive intervention. These cases highlight the importance of thorough counseling, clear risk–benefit discussions, and consideration of non-surgical alternatives to ensure ethically sound care.

Although outcomes were favorable across surgical approaches, differences in invasiveness, anatomical exposure, and technical complexity suggest that technique selection should be individualized. Open procedures may be appropriate for deep or adherent fragments, whereas endoscopic or limited dissection techniques may minimize tissue disruption in well-localized cases. In the absence of comparative outcome data, clinical judgment and procedural expertise remain central to selecting the optimal approach.

Beyond clinical considerations, retained catheter fragments may carry medicolegal implications, particularly in cases involving delayed recognition, neurological deterioration, or psychological distress. Notably, no cases in this review reported documented medicolegal claims or litigation related to retained catheter fragments. Clear documentation, timely disclosure, and structured follow-up are essential to support informed decision-making and minimize liability. Additionally, while surgical intervention is effective, it entails higher direct costs and resource use compared to conservative management. In this context, cost-effectiveness likely favors observation in asymptomatic patients, provided that appropriate surveillance mechanisms are in place. However, formal economic analyses are lacking and represent an important area for future research.

Finally, the identification of specific risk factors was not possible in this review. Although certain clinical and procedural variables appeared recurrent in individual cases, the available data were too limited and heterogeneous to allow for consistent or generalizable conclusions. As such, any potential associations remain speculative.

### 4.2. Strengths and Limitations

A key strength of this review is its patient-level synthesis, allowing for detailed assessment of clinical, procedural, and imaging factors. Standardized outcome measures enabled consistent comparisons across heterogeneous reports. Firth’s penalized logistic regression minimized small-sample bias, enhancing analytic reliability. Notably, the inclusion of a consensus-based matrix offers a novel, practical framework to guide decision-making in the absence of formal guidelines.

This review is significantly limited by its reliance on retrospective case reports and small series, which carry inherent biases. Favorable outcomes may be selectively reported, while complicated or unfavorable cases could be underrepresented, introducing potential publication bias. Furthermore, the lack of standardized follow-up durations or protocols across studies limits the ability to assess long-term outcomes or determine the true complication rate of retained catheter fragments. However, the patient-level approach enabled a granular analysis, which would be impossible in aggregate studies. Clinical management and outcome reporting were variable and often lacked standardization, but predefined outcome scales allowed for consistent comparisons. Imaging protocols and material details were inconsistently reported, yet objective measures, such as fragment length and detection thresholds supported meaningful evaluation. While Firth’s regression reduced small-sample bias, wide confidence intervals highlight the exploratory nature of the findings. Retrospective outcome grading based on narrative descriptions may introduce interpretation bias, though ambiguous cases were excluded to enhance reliability. Data were reconstructed from published reports, limiting detail, but broad inclusion improved the comprehensiveness of the review. Surgical decisions influenced by patient preference reflect real-world complexity, despite adding subjectivity. Finally, the consensus matrix synthesizes expert opinion rather than formal consensus, which should not be overinterpreted or used as a standard.

Given the rarity of retained epidural catheter fragments and their often unexpected or delayed presentation, prospective studies remain inherently difficult to conduct. The low incidence, lack of standardized diagnostic or management pathways, and variable follow-up further limit the feasibility of structured, high-quality prospective research. In contrast, collaborative multicenter registries may offer a more practical and effective approach, enabling systematic data collection across institutions, increasing case numbers, and ultimately improving the quality and generalizability of evidence in this field.

Despite these exploratory associations, it remains challenging to reliably identify definitive risk factors for retained catheter fragments. Given the rarity of this complication and the predominance of case reports in the existing literature, influencing factors remain speculative and are not supported by systematic data.

## 5. Conclusions

Retained epidural catheter fragments, though rare, pose a clinically significant challenge requiring nuanced management. Conservative treatment is frequently effective; however, symptom progression necessitating delayed surgery occurs in a substantial number of cases. The fragment length correlates with surgical intervention but lacks predictive value independent of catheter material, highlighting the primacy of anatomical context and composition over absolute size. The MRI offers superior sensitivity for localization and concurrent pathology assessment. When indicated, surgical retrieval is safe and consistently yields favorable outcomes. Optimal management is likely to benefit from individualized, evidence-informed decision-making that takes into account catheter material, imaging findings, and the evolving clinical course, though further prospective studies are needed to guide definitive treatment pathways.

## Figures and Tables

**Table 1:** Pooled patient characteristics of included studies.

Variable	Total (*n* = 51)	Conservative (*n* = 23)	Surgical (*n* = 28)	*p*-Value
Age—years	45.3 ± 21	45.3 ± 23.8	45.4 ± 17.9	0.99
Gender—no. (%)				
Female	33 (64.7)	18 (78.3)	15 (53.6)	0.08
Male	17 (33.3)	5 (21.7)	12 (42.9)	0.14
Missing	1 (2)	0 (0)	1 (3.6)	>0.99
Comorbidities—no. (%)				
Not reported	32 (62.7)	14 (60.9)	18 (64.3)	>0.99
Cardiovascular	7 (13.7)	4 (17.4)	3 (10.7)	0.69
GIT	3 (5.9)	2 (8.7)	1 (3.6)	0.59
Pulmonary	1 (2)	1 (4.3)	0 (0)	0.45
Musculoskeletal	7 (13.7)	3 (13)	4 (14.3)	>0.99
Oncological	5 (9.8)	4 (17.4)	1 (3.6)	0.16
Endocrinologic	4 (7.8)	3 (13)	1 (3.6)	0.32
Indication—no. (%)				
Orthopedic surgery	18 (35.3)	6 (26.1)	12 (42.9)	0.25
Delivery	20 (39.2)	11 (47.8)	9 (32.1)	0.39
GIT surgery	8 (15.7)	4 (17.4)	4 (14.3)	>0.99
Other	4 (7.8)	2 (8.7)	2 (7.1)	>0.99
Missing	1 (2)	0 (0)	1 (3.6)	>0.99
Catheter material—no. (%)				
**Polyamide (nylon)**	16 (31.4)	12 (52.2)	4 (14.3)	**0.06**
Polyurethane	8 (15.7)	2 (8.7)	6 (21.4)	0.27
Stainless steel	2 (3.9)	0 (0)	2 (7.1)	0.49
Polyethylene	2 (3.9)	0 (0)	2 (7.1)	0.49
Teflon	2 (3.9)	2 (8.7)	0 (0)	0.19
Missing	21 (41.2)	7 (30.4)	14 (50)	0.25
**Fragment length—mm**	82.1 ± 46.7	66.3 ± 49.7	103.3 ± 33.1	**0.02**
Spinal level—no. (%)				
T7/8	4 (7.8)	3 (13)	1 (3.6)	0.32
T8/9	2 (3.9)	1 (4.3)	1 (3.6)	>0.99
T9/10	1 (2)	1 (4.3)	0 (0)	0.45
T11/12	1 (2)	0 (0)	1 (3.6)	>0.99
T12/L1	2 (3.9)	1 (4.3)	1 (3.6)	>0.99
L2/L3	11 (21.6)	6 (26.1)	5 (17.9)	0.51
L3/L4	16 (31.4)	9 (39.1)	7 (25)	0.37
L4/L5	4 (7.8)	2 (8.7)	2 (7.1)	>0.99
L5/S1	1 (2)	0 (0)	1 (3.6)	>0.99
Sacral	2 (3.9)	0 (0)	2 (7.1)	0.49
Missing	7 (13.7)	0 (0)	7 (25)	0.01
McNab—no. (%)				
Excellent	43 (84.3)	20 (87)	23 (95.8) *	0.34
Good	4 (7.8)	3 (13)	1 (4.2) *	0.34
Missing	4 (7.8)	0 (0)	4 (14.3)	0.12
McCormick—no. (%)				
1	44 (86.3)	21 (91.3)	23 (95.8) *	0.34
2	3 (5.9)	2 (8.7)	1 (4.2) *	0.34
Missing	4 (7.8)	0 (0)	4 (14.3)	0.12
TDN—no. (%)				
**0**	12 (23.5)	12 (52.2)	0 (0)	**<0.0001**
2	2 (3.9)	2 (8.7)	0 (0)	0.19
**3**	37 (72.5)	9 (39.1)	28 (100)	**<0.0001**

Data are reported as means ± standard deviations or numbers (percentages). Statistically significant results (*p* < 0.05) were marked in bold font. Asterisk (*) indicates percentage values adjusted for missing data. Abbreviations: GIT, gastrointestinal tract; L, lumbar; *n*, absolute numbers; T, thoracal; TDN, Therapy–Disability–Neurology grade.

**Table 2:** Comparison of characteristics between cross-over and conservative patients.

Variable	Conservative (*n* = 14)	Cross-Over (*n* = 9)	*p*-Value
Age—years	46. ± 2.2	43.6 ± 21.3	0.78
Gender—female, no. (%)	11 (78.6)	7 (77.8)	>0.99
Comorbidities—no. (%)			
Not reported	9 (64.3)	5 (55.6)	>0.99
Cardiovascular	3 (21.4)	1 (11.1)	>0.99
GIT	1 (7.1)	1 (11.1)	>0.99
Pulmonary	1 (7.1)	0 (0)	>0.99
Musculoskeletal	1 (7.1)	2 (22.2)	0.54
Oncological	3 (21.4)	1 (11.1)	>0.99
Endocrinologic	2 (14.3)	1 (11.1)	>0.99
Indication—no. (%)			
Delivery	7 (50)	5 (55.6)	>0.99
GIT surgery	3 (21.4)	0 (0)	0.25
Orthopedics	2 (14.3)	4 (44.4)	0.16
Other	2 (14.3)	0 (0)	0.50
Spinal Level—no. (%)			
T7/8	3 (21.4)	0 (0)	0.25
T8/9	1 (7.1)	0 (0)	>0.99
T9/10	1 (7.1)	0 (0)	>0.99
T12/L1	0 (0)	1 (11.1)	0.39
L2/L3	3 (21.4)	3 (33.3)	0.64
L3/L4	5 (35.7)	4 (44.4)	>0.99
L4/L5	1 (7.1)	1 (11.1)	>0.99
Catheter material—no. (%)			
Polyamide (nylon)	11 (78.6)	1 (11.1)	**0.002**
Polyurethane	0 (0)	2 (22.2)	0.14
Teflon	1 (7.1)	1 (11.1)	>0.99
Missing	2 (14.3)	5 (55.6)	0.07
Fragment length—mm	50 ± 35.5	96.4 ± 60.5	**0.04**
TDN—no. (%)			
0	12 (85.7)	0 (0)	**<0.0001**
2	2 (14.3)	0 (0)	0.50
3	0 (0)	9 (100)	**<0.0001**
McNab—no. (%)			
Excellent	12 (85.7)	8 (88.9)	>0.99
Good	2 (14.3)	1 (11.1)	>0.99
McCormick—no. (%)			
1	12 (85.7)	8 (88.9)	>0.99
2	2 (14.3)	1 (11.1)	>0.99

Data are reported as means ± standard deviations or numbers (percentages). Statistically significant results (*p* < 0.05) were marked in bold font. Abbreviations: GIT, gastrointestinal tract; L, lumbar; mm, millimeter; *n*, absolute number; T, thoracal; TDN, Therapy–Disability–Neurology grade.

**Table 3:** Differences between surgical subgroups.

Variable	Open (*n* = 13)	Endoscopic (*n* = 12)	Limited Open Dissection (*n* = 12)	*p*-Value
Age—years	50.8 ± 20.1	36.2 ± 18.8	41.5 ± 15.9	0.27
Gender—no. (%)				
Female	8 (61.5)	7 (58.3)	7 (58.3)	>0.99
Male	5 (38.5)	5 (41.7)	4 (33.3)	>0.99
Missing	0 (0)	0 (0)	1 (8.3)	0.65
Comorbidities—no. (%)				
Not reported	6 (46.2)	8 (66.7)	9 (75)	0.33
Cardiovascular	3 (23.1)	1 (8.3)	0 (0)	0.29
GIT	1 (7.7)	0 (0)	1 (8.3)	>0.99
Musculoskeletal	5 (38.5)	1 (8.3)	1 (8.3)	0.17
Oncological	0 (0)	1 (8.3)	1 (8.3)	0.53
Endocrinologic	1 (7.7)	1 (8.3)	0 (0)	>0.99
Indication—no. (%)				
Orthopedic surgery	7 (53.8)	4 (33.3)	5 (41.7)	0.65
Delivery	4 (30.8)	5 (41.7)	4 (33.3)	0.91
GIT surgery	1 (7.7)	2 (16.7)	2 (16.7)	0.72
Other	1 (7.7)	1 (8.3)	0 (0)	>0.99
Missing	0 (0)	0 (0)	1 (8.3)	0.65
Catheter material—no. (%)				
Polyurethane	3 (23.1)	1 (8.3)	4 (33.3)	0.38
Polyamide (nylon)	3 (23.1)	0 (0)	2 (16.7)	0.33
Polyethylene	1 (7.7)	0 (0)	1 (8.3)	>0.99
Stainless steel	1 (7.7)	1 (8.3)	0 (0)	>0.99
Teflon	1 (7.7)	0 (0)	0 (0)	>0.99
Not specified	4 (30.8)	10 (83.3)	5 (41.7)	0.02
Fragment length—mm	101 ± 46	66.7 ± 25.2	112.7 ± 39.5	0.24
Spinal level—no. (%)				
T7/8	0 (0)	0 (0)	1 (8.3)	0.65
T8/9	0 (0)	0 (0)	1 (8.3)	0.65
T11/12	0 (0)	0 (0)	1 (8.3)	0.65
T12/L1	1 (7.7)	1 (8.3)	0 (0)	>0.99
L2/L3	5 (38.5)	1 (8.3)	2 (16.7)	0.22
L3/L4	5 (38.5)	1 (8.3)	5 (41.7)	0.15
L4/L5	2 (15.4)	1 (8.3)	0 (0)	0.76
L5/S1	0 (0)	1 (8.3)	0 (0)	0.65
Sacral	0 (0)	0 (0)	2 (16.7)	0.19
Missing	0 (0)	7 (58.3)	0 (0)	0.0002
Cross-over patients—no. (%)	5 (38.5)	2 (16.7)	2 (16.7)	0.44
McNab—no. (%)				
Excellent	11 (84.6)	12 (100)	8 (100) *	0.33
Good	2 (15.4)	0 (0)	0 (0)	0.32
Missing	0 (0)	0 (0)	4 (33.3)	0.02
McCormick—no. (%)				
1	12 (100)	12 (100)	8 (100) *	>0.99
2	1 (7.7)	0 (0)	0 (0)	>0.99
Missing	0 (0)	0 (0)	4 (33.3)	0.02

Data are reported as means ± standard deviations or numbers (percentages). Statistically significant results (*p* < 0.05) were marked in bold font. Asterisk (*) indicates percentage values adjusted for missing data. Abbreviations: GIT, gastrointestinal tract; L, lumbar; mm, millimeter; *n*, absolute number; T, thoracal.

**Table 4:** Correlations of factors associated with receiving surgery.

	Univariate			Multivariate		
Variable	OR	95% CI	*p*-Value	OR	95% CI	*p*-Value
Age	1.00	0.97–1.03	0.80	1.04	0.97–1.14	0.31
Gender	0.45	0.09–1.82	0.29	0.05	0.00–0.86	0.08
**Material**	2.49	1.084–9.00	**0.03**	1.65	0.66–5.66	0.33
Level	2.08	1.00–4.67	0.06	7.00	1.14–134.9	0.09
Fragment length *	1.03	1.01–1.06	**0.0006**	1.05	1.01–1.10	**0.04**

Data is presented as odds ratios with corresponding 95% CI and *p*-values for univariate and multivariate logistic regression, respectively. Statistically significant results (*p* < 0.05) were marked in bold font. Abbreviations: CI, confidence interval; OR, odds ratio. * The OR for each 1 mm increase in catheter length is calculated.

**Table 5:** Author-derived consensus matrix on the management of retained epidural catheter fragments.

Domain	Consensus Statement	Agreement Level	Rationale	Supporting Authors	Opposing Viewpoints	Scientific Uncertainty/Notes
Documentation	Thorough documentation of retained fragments and clear communication with patients and healthcare providers, accompanied by regular follow-up.	Strong Consensus	Ensuring patient safety, reducing anxiety, and managing potential late complications	[4,22,25,27,30,31,32,42,44,45]	-	Crucial for medico-legal and patient safety purposes.
Imaging	MRI is recommended as the primary imaging modality to localize retained catheter fragments; CT scans may complement MRI, particularly in detecting small fragments or when MRI is inconclusive.	Strong Consensus	Superior sensitivity and specificity; enhanced fragment visualization	[2,4,22,30,32,44,48,49,52]	[5,42]	CT beneficial when MRI inconclusive or unavailable; CAVE: retained metal coil
Management Strategy	Conservative management is acceptable for asymptomatic patients, provided there is thorough patient education and regular follow-up. Surgical intervention is recommended for symptomatic patients or those at significant risk of complications.	Strong Consensus	Low risk for asymptomatic cases; inert catheter material	[2,4,5,22,25,27,29,30,39,44,45,51,52]	[38] (aggressive surgery even if asymptomatic), [7,31,41] (early proactive surgery)	Regular follow-up essential for asymptomatic patients
Criteria for Surgery	Surgery is indicated in symptomatic patients, presence of neurological symptoms, persistent cerebrospinal fluid leakage, intrathecal fragments, infection risk, and catheter fragments protruding through the skin.	Moderate Consensus	Prevention of severe complications: neurological deficits, infection, hematoma risk	[2,7,22,29,30,31,39,44,49,51,52]	[4,5,25,27,42] (surgery only if symptomatic)	Individual risk assessment essential; variable opinions on fragment length as sole criterion
Timing of Surgery	Early surgical removal (within days to weeks) recommended to prevent adhesions, fibrosis, or complicated delayed surgeries.	Moderate Consensus	Minimization of fibrosis, adhesions, granulation tissue	[2,7,29,31]	[4,22] (only if complications arise)	Optimal surgical window varies; early intervention generally advised
Follow-Up	Annual follow-up recommended for asymptomatic patients, with closer intervals based on individual risk assessment.	Limited Evidence and Consensus	Detection of late-onset complications	[22,25,30,51]	-	Precise optimal interval uncertain; individualized based on patient risk factors

Abbreviations: CT, computed tomography; MRI, magnetic resonance imaging.

## Data Availability

The raw data supporting the conclusions of this article will be made available by the authors on request. The datasets generated and analyzed during the current study are available from the corresponding author on reasonable request. All data supporting the findings of this review were extracted from publicly available sources such as peer-reviewed articles.

## Supplementary Materials

The following supporting information can be downloaded at https://www.mdpi.com/article/10.3390/jcm14124265/s1.

## Abbreviations

The following abbreviations are used in this manuscript:

AUC	Area Under the Curve
BMI	Body Mass Index
CI	Confidence Interval
GRADE	Grading of Recommendations Assessment, Development, and Evaluation
ICMJE	International Committee of Medical Journal Editors
JBI	Joanna Briggs Institute
MeSH	Medical Subject Headings
MRI	Magnetic Resonance Imaging
OR	Odds Ratio
PICOS	Population, Intervention, Comparison, Outcomes, and Study
PRISMA	Preferred Reporting Items for Systematic reviews and Meta-Analyses
PROSPERO	International Prospective Register of Systematic Reviews
ROC	Receiver Operating Characteristic
TDN	Therapy–Disability–Neurology score
UBE	Unilateral Biportal Endoscopy
US	Ultrasound
X-ray	Radiography
