# Supplementary material for: Management of Retained Epidural Catheter Fragments: A Narrative Review of Individual Patient Data
Source: J Clin Med. 2025 Jun 16;14(12):4265. doi: 10.3390/jcm14124265 (PMC12193838; doi:10.3390/jcm14124265)
Supplement: Supplementary file 1 [file jcm-14-04265-s001.zip › jcm-3649502-supplementary.pdf]

Supplementary Material

Table of Contents

Table S1. PRISMA Checklist. 2

Table S2. PICOS framework for inclusion and exclusion criteria. 4

Table S3. Inclusion and exclusion criteria. 5

Table S4. Search strategy. 6

Table S5. Quality assessment by the JBI tool of included case reports. 7

Table S6. Overview of Included Studies. 9

Table S7. Leave-one-out sensitivity analysis of firth logistic regression coefficients 11

Table S8. Firth Penalized Logistic Regression for Predictors of Surgical Intervention 12

References 13

Table S1. PRISMA Checklist.

Section and Topic	Item #	Checklist item	Location where item is reported
**TITLE**			
Title	1	Identify the report as a systematic review.	1
**ABSTRACT**			
Abstract	2	See the PRISMA 2020 for Abstracts checklist.	1
**INTRODUCTION**			
Rationale	3	Describe the rationale for the review in the context of existing knowledge.	2
Objectives	4	Provide an explicit statement of the objective(s) or question(s) the review addresses.	2
**METHODS**			
Eligibility criteria	5	Specify the inclusion and exclusion criteria for the review and how studies were grouped for the syntheses.	3
Information sources	6	Specify all databases, registers, websites, organisations, reference lists and other sources searched or consulted to identify studies. Specify the date when each source was last searched or consulted.	3-4
Search strategy	7	Present the full search strategies for all databases, registers and websites, including any filters and limits used.	4
Selection process	8	Specify the methods used to decide whether a study met the inclusion criteria of the review, including how many reviewers screened each record and each report retrieved, whether they worked independently, and if applicable, details of automation tools used in the process.	4
Data collection process	9	Specify the methods used to collect data from reports, including how many reviewers collected data from each report, whether they worked independently, any processes for obtaining or confirming data from study investigators, and if applicable, details of automation tools used in the process.	4
Data items	10a	List and define all outcomes for which data were sought. Specify whether all results that were compatible with each outcome domain in each study were sought (e.g. for all measures, time points, analyses), and if not, the methods used to decide which results to collect.	4
	10b	List and define all other variables for which data were sought (e.g. participant and intervention characteristics, funding sources). Describe any assumptions made about any missing or unclear information.	4
Study risk of bias assessment	11	Specify the methods used to assess risk of bias in the included studies, including details of the tool(s) used, how many reviewers assessed each study and whether they worked independently, and if applicable, details of automation tools used in the process.	5
Effect measures	12	Specify for each outcome the effect measure(s) (e.g. risk ratio, mean difference) used in the synthesis or presentation of results.	5
Synthesis methods	13a	Describe the processes used to decide which studies were eligible for each synthesis (e.g. tabulating the study intervention characteristics and comparing against the planned groups for each synthesis (item #5)).	5-6
	13b	Describe any methods required to prepare the data for presentation or synthesis, such as handling of missing summary statistics, or data conversions.	5
	13c	Describe any methods used to tabulate or visually display results of individual studies and syntheses.	5
	13d	Describe any methods used to synthesize results and provide a rationale for the choice(s). If meta-analysis was performed, describe the model(s), method(s) to identify the presence and extent of statistical heterogeneity, and software package(s) used.	5
	13e	Describe any methods used to explore possible causes of heterogeneity among study results (e.g. subgroup analysis, meta-regression).	6
	13f	Describe any sensitivity analyses conducted to assess robustness of the synthesized results.	6
Reporting bias assessment	14	Describe any methods used to assess risk of bias due to missing results in a synthesis (arising from reporting biases).	4
Certainty assessment	15	Describe any methods used to assess certainty (or confidence) in the body of evidence for an outcome.	4
**RESULTS**			
Study selection	16a	Describe the results of the search and selection process, from the number of records identified in the search to the number of studies included in the review, ideally using a flow diagram.	6
	16b	Cite studies that might appear to meet the inclusion criteria, but which were excluded, and explain why they were excluded.	6
Study characteristics	17	Cite each included study and present its characteristics.	6-8
Risk of bias in studies	18	Present assessments of risk of bias for each included study.	8
Results of individual studies	19	For all outcomes, present, for each study: (a) summary statistics for each group (where appropriate) and (b) an effect estimate and its precision (e.g. confidence/credible interval), ideally using structured tables or plots.	8-16
Results of syntheses	20a	For each synthesis, briefly summarise the characteristics and risk of bias among contributing studies.	8-16
	20b	Present results of all statistical syntheses conducted. If meta-analysis was done, present for each the summary estimate and its precision (e.g. confidence/credible interval) and measures of statistical heterogeneity. If comparing groups, describe the direction of the effect.	8-16
	20c	Present results of all investigations of possible causes of heterogeneity among study results.	8-16
	20d	Present results of all sensitivity analyses conducted to assess the robustness of the synthesized results.	15
Reporting biases	21	Present assessments of risk of bias due to missing results (arising from reporting biases) for each synthesis assessed.	8
Certainty of evidence	22	Present assessments of certainty (or confidence) in the body of evidence for each outcome assessed.	8-16
**DISCUSSION**			
Discussion	23a	Provide a general interpretation of the results in the context of other evidence.	17-18
	23b	Discuss any limitations of the evidence included in the review.	18
	23c	Discuss any limitations of the review processes used.	18
	23d	Discuss implications of the results for practice, policy, and future research.	18
**OTHER INFORMATION**			
Registration and protocol	24a	Provide registration information for the review, including register name and registration number, or state that the review was not registered.	2
	24b	Indicate where the review protocol can be accessed, or state that a protocol was not prepared.	2
	24c	Describe and explain any amendments to information provided at registration or in the protocol.	2
Support	25	Describe sources of financial or non-financial support for the review, and the role of the funders or sponsors in the review.	1
Competing interests	26	Declare any competing interests of review authors.	1

Section and Topic	Item #	Checklist item	Location where item is reported
Availability of data, code and other materials	27	Report which of the following are publicly available and where they can be found: template data collection forms; data extracted from included studies; data used for all analyses; analytic code; any other materials used in the review.	n.a.

Table S2. PICOS framework for inclusion and exclusion criteria**.**

Category	Description	Inclusion	Exclusion
**Population**	Adult patients (≥18 years) who experienced disrupted epidural catheters requiring surgical retrieval or conservative management.	Studies focusing on adult patients with epidural catheter breakage, detailing retrieval or conservative management strategies, and reporting outcomes such as success rates, safety, and complications.	Pediatric populations; cases involving non-epidural catheters; catheter removal without surgical intervention.
**Intervention**	Surgical retrieval methods for broken epidural catheters, including open surgery (laminectomy, laminotomy), minimally invasive and endoscopic procedures, fluoroscopy-guided techniques, and tissue dissection.	Studies clearly describing surgical removal techniques (open, minimally invasive, endoscopic, fluoroscopy-guided, or tissue dissection).	Studies focusing exclusively on prevention or lacking surgical intervention details.
**Comparison**	Conservative management approaches, including observation and serial imaging.	Studies providing detailed conservative management strategies (observation or other non-surgical interventions).	Studies without a clearly described conservative management approach.
**Outcome**	Success rates of catheter retrieval, complications (neurological deficits, infections), patient recovery, and long-term follow-up outcomes.	Studies explicitly reporting outcomes on retrieval success, complications, patient recovery, and long-term prognosis.	Studies lacking detailed outcome data or not reporting specific outcomes related to retrieval success or patient prognosis.
**Study Design**	Original research articles including randomized controlled trials, cohort studies, case-control studies, and comprehensive case series.	Peer-reviewed original research published in English reporting primary data on surgical retrieval of disrupted epidural catheters.	Review articles, meta-analyses, studies lacking primary data, and non-English publications without available translations.

Table S3. Inclusion and exclusion criteria.

Inclusion criteria	Exclusion criteria
• Published in English language<br>• Peer-reviewed journal articles<br>• Original research articles providing primary data on disrupted epidural catheters requiring retrieval or conservative management<br>• Studies involving adult populations (≥18 years)	• Publications in languages other than English<br>• Editorials, letters, commentaries, books, or book chapters, animal studies or lab-based studies<br>• Studies not reporting specific and measurable clinical outcomes (success rates, complications, neurological outcomes)<br>• Animal studies, laboratory studies, or pre-clinical research<br>• Studies including pediatric or adolescent populations (<18 years)

Table S4. Search strategy.

Database	Search Date	Time frame	Search Syntax	Hits (n)
Google Scholar	15-Mar-2025	No restrictions	"retained epidural catheter" OR "fractured epidural catheter" OR "ruptured epidural catheter" OR "broken epidural catheter" OR "migrated epidural catheter" OR "dislodged epidural catheter"	n = 312
ScienceDirect	15-Mar-2025	No restrictions	"retained epidural catheter" OR "fractured epidural catheter" OR "ruptured epidural catheter" OR "broken epidural catheter" AND ("surgical removal" OR "surgical extraction" OR "conservative management" OR "non-surgical treatment")	n = 36
Web of Science	15-Mar-2025	No restrictions	"retained epidural catheter" OR "fractured epidural catheter" OR "ruptured epidural catheter" OR "broken epidural catheter" AND ("surgical removal" OR "surgical extraction" OR "conservative management" OR "non-surgical treatment")	n = 77
PubMed	15-Mar-2025	No restrictions	"retained epidural catheter" OR "fractured epidural catheter" OR "ruptured epidural catheter" OR "broken epidural catheter" OR "migrated epidural catheter" OR "dislodged epidural catheter" AND ("surgical removal" OR "surgical extraction" OR "conservative management" OR "non-surgical treatment")	n = 14
BASE	15-Mar-2025	No restrictions	"retained epidural catheter" OR "fractured epidural catheter" OR "ruptured epidural catheter" OR "broken epidural catheter" AND ("surgical removal" OR "surgical extraction" OR "conservative management" OR "non-surgical treatment")	n = 7
Cochrane Library	15-Mar-2025	No restrictions	"retained epidural catheter" OR "fractured epidural catheter" OR "ruptured epidural catheter" OR "broken epidural catheter"	n = 2

Table S5. Quality assessment by the JBI tool of included case reports.

Study	Q1	Q2	Q3	Q4	Q5	Q6	Q7	Q8
Chun and Karp, 1966	+	+	+	+	-	-	+	-
Blass, Roberts, and Wiley, 1981	+	+	+	+	+	+	+	+
Manchikanti and Bakhit, 1997	+	+	+	+	+	+	+	+
Nishio et al., 2001	-	+	+	+	+	+	+	+
Asai et al., 2001	+	+	+	+	+	+	+	+
Ugboma et al., 2002	+	+	+	+	+	+	+	+
Lee et al., 2010	+	+	+	+	?	+	+	+
Pinciroli and Fumagalli, 2015	+	+	+	+	+	+	+	+
Kim, Shin and Lee, 2016	+	+	+	+	+	+	+	+
Hippalgaonkar et al. 2017	+	+	+	+	+	+	+	+
Powers and Elmofty, 2020	+	?	+	+	+	+	+	+
Patel et al., 2021	+	+	?	+	+	+	+	+
Taksande et al., 2021	+	+	+	?	+	+	+	+
Walia et al., 2022	-	?	?	-	+	+	+	+
Sulhan et al., 2022	+	+	+	+	+	?	+	+
Gombels, Rusby and Slater, 2022	+	+	+	+	+	+	+	+
Kumar et al., 2023	+	+	?	+	+	+	+	+
Motov and Stienen, 2024	+	?	+	+	+	+	-	+
Alfadhel et al., 2024	+	+	+	+	+	+	+	+
Subith et al., 2024	+	+	+	+	+	+	+	+
Tio, Macmurdo and McKenzie et al., 1979	+	+	+	+	+	+	+	+
Moerman, Porcelijin and Deen, 1980	+	+	+	-	-	-	+	+
Staats, Stinson and Lee, 1995	+	+	+	+	+	+	+	+
Blanchard et al., 1997	+	+	+	+	+	+	+	+
Collier, 2000	+	+	+	+	+	+	+	+
Schummer and Schummer, 2002	+	?	+	-	?	+	+	+
Demiraran, Yucel and Erdogmus, 2006	+	+	+	+	+	+	+	+

Pant et al., 2007	+	+	+	+	+	+	+	+
Rajendra and Popham, 2008	+	+	+	+	+	+	+	+
Eap et al., 2010	-	+	-	+	+	+	+	+
Drake, 2012	+	-	+	+	-	+	-	+
Abouhashem, 2013	+	+	+	+	+	+	-	+
Üşar et al.,2015	+	+	+	+	+	+	+	+
Tarukado et al., 2015	+	+	+	+	+	+	+	+
Ishikawa et al., 2016	+	+	+	+	+	+	+	+
Reena and Vikram, 2017	+	+	+	+	+	+	+	+
Siddappa, Kim and Khandge, 2020	+	+	+	+	+	+	+	+
Jiménez-Ponce et al., 2023	+	+	+	+	+	+	+	+

Abbreviations: Q1 = Were the patient's demographic characteristics clearly described?; Q2 = Was the patient's history clearly described and presented as a timeline?; Q3 = Was the current clinical condition of the patient on presentation clearly described?; Q4 = Were diagnostic tests or assessment methods and the results clearly described?; Q5 = Was the intervention(s) or treatment procedure(s) clearly described?; Q6 = Was the post-intervention clinical condition clearly described?; Q7 = Were adverse events (harms) or unanticipated events identified and described?; Q8 = Does the case report provide takeaway lessons? Not Hösslin et al. and Kong et al. + = yes, - = no, ? = unclear

Table S6. Overview of Included Studies.

Author	Group	Patients (n)	Age	Gender	Comorbidities	Catheter Material	Indication Catheter Placement	Spinal level	Imaging	Surgical Approach	Fragment Length (mm)	Cause of catheter failure	Complications	TDN	McCormick	MacNab
Chun and Karp, 1966 [1]	S	1	25	F	-	-	Labour	Sacral hiatus	-	Tissue dissection	102	-	None	T3 D1 N1	-	-
Chun and Karp, 1966 [1]	S	1	18	F	-	Polyethylene	Labour	Sacral hiatus	-	Tissue dissection	152	Shearing of looped catheter when grapsed with forceps	None	T3 D1 N1	-	-
Blass, Roberts, and Wiley, 1981 [2]	S	1	24	F	-	-	Labour	L3-L4	-	Laminectomy	-	Knot around strand of ligamentum flavum	None	T3 D1 N1	1	Excellent
Manchikanti and Bakhit, 1997 [3]	S	1	68	M	Lumbar epidural fibrosis	Stainless steel	Low back pain with radiculopathy management	L5-S1	X-ray	Endoscopic	70	Multiple redirection and rotations, pulling out needle and catheter together under resistance	None	T3 D1 N1	1	Excellent
Nishio et al., 2001 [4]	S	1	48	M	-	Polyurethane	Surgical analgesia for internal tibia fracture fixation	L2–L3	X-ray	-	-	Microdamage to the catheters by gripping with stainless steel hemostat	None	T3 D1 N1	1	Excellent
Asai et al., 2001 [5]	S	1	57	F	-	Polyurethane	Total knee arthroplasty	L3-L4	-	Tissue dissection	80	-	None	T3 D1 N1	-	-
Ugboma et al., 2002 [6]	S	1	74	F	Complete AV-block, TIAs, embolic stroke, osteoporosis, severe osteoarthritis	Polyurethane	Total hip replacement	L3-L4	X-ray, CT	Laminectomy	90	Removal despite resistance	Intraoperative CSF leak from catheter (sealed intraoperatively)	T3 D1 N1	1	Excellent
Lee et al., 2010 [7]	S	1	70	M	Esophageal cancer	Polyurethane	Esophagectomy	T7-T8	X-ray	Tissue dissection	-	Catheter broke when gasped by kelly clamp	None	T3 D1 N1	1	Excellent
Pinciroli and Fumagalli, 2015 [8]	S	1	53	F	Diffuse right and left colonic diverticular disease	Polyurethane	Painful rib syndrome during pregnancy 12 years before	T11-12	X-ray	Tissue dissection	150	-	None	T3 D1 N1	1	Excellent
Kim, Shin and Lee, 2016 [9]	S	1	68	M	Severe degenerative central canal stenosis, bilateral foraminal stenosis, degenerative spondylolisthesis	Stainless steel	Percutaneous epidural neuroplasty for neurogenic intermittent claudication	L4-L5	CT	Laminectomy with spondylodesis	120	Removal despite resistance	None	T3 D1 N1	1	Excellent
Hippalgaonkar et al. 2017 [10]	S	1	68	M	Hypertension	Polyamide (nylon)	Bilateral inguinal hernioplasty	L3-L4	X-ray, CT	Laminectomy	170	-	None	T3 D1 N1	1	Excellent
Powers and Elmofty, 2020 [11]	S	1	-	-	Baastrup Disease	-	-	L3-L4	-	-	-	-	None	T3 D1 N1	-	-
Patel et al., 2021 [12]	S	1	52	F	-	Polyamide (nylon)	Liver transplant (donor) surgery	T8-T9	X-ray,CT, MRI	Tissue dissection	120	Heavy contact between the tip of epidural needle and bony surface, simultaneous removal of catheter and needle	None	T3 D1 N1	1	Excellent
Taksande et al., 2021 [13]	S	1	47	M	-	Polyamide (nylon)	Knee surgery	L2-L3	X-ray	Laminectomy	75	Catheter and needle were simultaneously removed	None	T3 D1 N1	1	Excellent
Walia et al., 2022 [14]	S	1	30	M	Avascular necrosis of the hip	Polyethylene	Total hip replacement	L2-3	X-ray	Laminotomy	-	Catheter and needle were simultaneously removed	Back pain persisted for six weeks after surgery	T3 D1 N1	2	Good
Sulhan et al., 2022 [15]	S	1	39	F	Moya Moya ischemic cerebrovascular disease	-	Craniotomy for Shunt	L3-4	CT	Endoscopic	-	-	spinal headache, which resolved after epidural blood patch administration	T3 D1 N1	1	Excellent
Kong et al., 2022 [16]	S	7	**45,43***	0, 0, 0, 1, 1, 1, 1	-	-	anesthesia (3x cesarian section, 2x lower extremity surgery, 2x pelvic surgery	-	X-ray, CT, MRI	Endoscopic	-	3x rupture during catheterization, 2x needle retraction, 1 during extubation	None	T3 D1 N1	1	Excellent
Gombels, Rusby and Slater, 2022 [17]	S	1	37	F	-	-	Labour	L3-L4	CT, MRI	Tissue dissection	110	Removal despite resistance	None	T3 D1 N1	1	Excellent
Kumar et al., 2023 [18]	S	1	39	M	-	-	Tibia reconstruction surgery	L2–L3	X-ray	Tissue dissection	80	Excessive force in pulling off catheter simultaneously through a needle	None	T3 D1 N1	1	Excellent
Motov and Stienen, 2024 [19]	S	1	26	F	Hypothyroidism, Factor-V Leiden	-	Labour	Th12-L1	X-ray, CT, MRI	Endoscopic	90	-	None	T3 D1 N1	1	Excellent
Alfadhel et al., 2024 [20]	S	1	23	F	-	Polyurethane	Labour	L2-L3	X-ray, CT, MRI	Laminectomy	60	Looped catheter was pulled out slow after flushing with saline	None	T3 D1 N1	1	Excellent
Subith et al., 2024 [21]	S	1	41	M	-	Polyamide (nylon)	Tibia material removal	L4-L5	X-ray, CT, MRI	Laminectomy	80	Multiple insertion attempts	None	T3 D1 N1	1	Excellent
Tio, Macmurdo and McKenzie et al., 1979 [22]	C	1	18	F	-	Teflon	Labour	L2-L3	X-ray	-	80	Shredding by the tuohy needly	None	-	1	Excellent

Moerman, Porcelijin and Deen, 1980 [23]	C	1	24	M	-	-	Knee meniscecto my	L3-L4	X-ray	-	10	-	None	-	1	Excell ent
Staats, Stinson and Lee, 1995 [24]	C	1	64	F	-	Teflon	Total hip replacement	L3-L4	MRI, CT	Laminecto my	15	Manipulation of catheter	None	T3 D1 N1	1	Excell ent
Blanchard et al., 1997 [25]	C	1	34	F	-	-	Cesarian section	L3-L4	X-ray, CT, MRI	Laminecto my	150	Multiple loops with excessive force	None	T3 D1 N1	1	Excell ent
Collier, 2000 [26]	C	1	31	F	-	Polyamide (nylon)	Labour	L4-L5	X-ray, MRI, CT	-	50	Withdrew through tuohy needle	Backpain, paresthesia, sensory loss	T2 D1 N2	2	Good
Collier, 2000 [26]	C	1	20	F	-	Polyamide (nylon)	Labour	L2-L3	X-ray, MRI, CT	-	40	Withdrew through tuohy needle	Backpain, hypesthesia	T2 D1 N2	2	Good
Schummer and Schummer, 2002 [27]	C	1	20	F	-	Polyamide (nylon)	Labour	L3-L4	CT, MRI	-	30	-	None	-	1	Excell ent
Demiraran, Yucel and Erdogmus, 2006 [28]	C	1	25	M	-	Polyamide (nylon)	Tibia fracture surgery	L3-L4	US	Tissue dissection	50	Untrained personal	None	T3 D1 N1	1	Excell ent
Pant et al., 2007 [29]	C	1	70	M	COPD, rib fractures	Polyamide (nylon)	Rib fracture pain managemen t	T8-T9	CT, MRI	-	60	-	None	-	1	Excell ent
Rajendra and Popham, 2008 [30]	C	1	28	F	-	Polyamide (nylon)	Cesarian section	L3-L4	X-ray, CT	-	100	Withdrew through tuohy needle	None	-	1	Excell ent
Eap et al., 2010 [31]	C	1	24	F	-	-	Labour	L2-L3	X-ray, CT	Endoscopi c	-	Withdrew through tuohy needle	None	T3 D1 N1	1	Excell ent
Drake, 2012 [32]	C	1	-	F	-	Polyamide (nylon)	Labour	L2-L3	X-ray, MRI	-	-	Mishandling during catheter insertion	None	-	1	Excell ent
Abouhashem, 2013 [33]	C	1	33	F	-	-	Labour	L3-L4	X-ray	Tissue dissection	170	-	None	T3 D1 N1	1	Excell ent
Üşar et al.,2015 [34]	C	1	31	F	-	Polyamide (nylon)	Labour	L3-L4	-	-	20	Suspected cut by the Tuohy needle during insertion	None	-	1	Excell ent
Tarukado et al., 2015 [35]	C	1	82	F	Osteoarthros is	Polyurethane	Total knee arthroplasty	L2-L3	X-ray, CT	Laminecto my	130	Catheter kinking on the surface of the dura mater,adhesion, Baastrup disease	None	T3 D1 N1	1	Excell ent
von Hösslin et al.,2016 [36]	C	1	91	F	Acute renal insufficiency , hypertensio n, osteoporosis, hiatal hernia, peripheral arterial occlusive disease	Polyamide (nylon)	Low anterior resection (colon diverticuliti s with stenosis)	T9-10	-	-	80	-	None	T3 D1 N1	1	Excell ent
von Hösslin et al.,2016 [36]	C	1	69	M	Coronary heart disease, arterial hypertensio n, diabetes mellitus	Polyamide (nylon)	Partial right pneumecto my (sarcoma relapse)	T7-8	-	-	15	-	None	T3 D1 N1	1	Excell ent
von Hösslin et al.,2016 [36]	C	1	67	F	Ileus and cachexia due to metastasisin g rectal carcinoma, depression, chronic pain	Polyamide (nylon)	Laparotomy (ileus due to rectal cancer)	T7-8	-	-	30	Cutoff while removal	None	T3 D1 N1	1	Excell ent
von Hösslin et al.,2016 [36]	C	1	81	F	Hypertensio n, diabetes mellitus	Polyamide (nylon)	Gastric resection (Gastrointes tinal stroma tumour)	T7-8	-	-	120	-	None	T3 D1 N1	1	Excell ent
Ishikawa et al., 2016 [37]	C	1	64	F	Arterial hypertensio n, rheumatoid arthritis, arryhthmmi a, ileal conduit diversion	-	Ileal condoit surgery (18 years prior)	T12-L1	X-ray, CT, MRI, US	Laminecto my	-	-	Spinal cord subarachnoid and subdural hematoma	T3 D1 N2	1	Good
Reena and Vikram, 2017 [38]	C	1	55	F	-	-	Hysterecto my	L3-L5	X-ray	-	15	Relocation of needle	None	-	1	Excell ent
Siddappa, Kim and Khandge, 2020 [39]	C	1	24	M	Acute lymphoblast ic leukemia (10-year history), L4 lymphoma and L4-L5 lumbar disc herniation	Polyurethane	Pain managemen t disc herniation	L4-L5	X-ray, CT	Endoscopi c	40	Removal despite resistance	None	T3 D1 N1	1	Excell ent
Jiménez-Ponce et al., 2023 [40]	C	1	42	F	Hypothyroi dism	-	Cesarian section	L2-L3	X-ray, MRI	Laminecto my	120	Coiling in an S-shape and trapped between facet joint	None	T3 D1 N1	1	Excell ent

Table S7. Leave-one-out sensitivity analysis of firth logistic regression coefficients.

Predictor	Min Coefficient	Max Coefficient	Mean Coefficient	Standard Deviation
Intercept	-3.6760	0.6234	-2.5540	0.8088
Age	-0.0458	0.0365	0.0190	0.0145
Gender (female)	-2.9314	-1.4912	-1.9601	0.2818
Polyurethane	2.0699	4.5734	2.7627	0.4159
Polyethylene	-3.2646	4.1853	0.3265	1.2384
Stainless steel	-1.8790	1.2768	-1.1762	0.6088
Teflon	-0.6652	6.1924	1.3092	0.8080
Lumbar	-0.4472	3.2226	1.6734	0.5535
Lumbosacral	0.0000	3.4344	2.3485	0.7201
Fragment Length	-2.7683	0.0393	-0.0454	0.4152

Table S8. Firth Penalized Logistic Regression for Predictors of Surgical Intervention

Variable	Univariate			Multivariate		
	OR	95% CI	p-value	OR	95% CI	p-value
Polyurethane	35.6	3.37 – 4913.47	**0.0011**	14.47	1.07 – 1883.10	**0.044**
Polyethylene	10.45	0.70 – 1540.93	0.093	1.33	0.0006 – 3100.00	0.940
Stainless Steel	10.45	0.70 – 1540.93	0.093	0.25	0.00018 – 88.08	0.615
Teflon	2.09	0.14 – 30.69	0.562	3.49	0.165 – 105.60	0.403
Fragment Length	1.03	1.01 – 1.06	**0.0011**	1.017	0.989 – 1.064	0.275
Age	1.00	0.97 – 1.03	0.789	1.02	0.96 – 1.12	0.527
Thoracolumbar	7.86	0.45 – 1223.85	0.168	—	—	—
Lumbar	3.72	0.82 – 19.15	0.089	5.84	0.21 – 1191.50	0.308
Lumbosacral	11.00	0.72 – 1665.70	0.088	13.39	0.019 – 44834.30	0.418
Gender	0.49	0.11 – 1.88	0.306	0.15	0.007 – 1.41	0.098

References

[1] Chun, L.; Karp, M. Unusual complications from placement of catheters in caudal canal in obstetrical anesthesia. *Anesthesiology*, **1966**, *27,* 96–97.

[2] Blass, N.H.; Roberts, R.B.; Wiley, J.K. The case of the errant epidural catheter. *Anesthesiology*, **1981**, *54,* 419–421.

[3] Manchikanti, L.; Bakhit, C.E. Removal of a torn Racz catheter from lumbar epidural space. *Regional anesthesia*, **1997**, *22,* 579–581.

[4] Nishio, I.; Sekiguchi, M.; Aoyama, Y.; Asano, S.; Ono, A. Decreased tensile strength of an epidural catheter during its removal by grasping with a hemostat. *Anesthesia and analgesia*, **2001**, *93,* 210-2, TOC.

[5] Asai, T.; Yamamoto, K.; Hirose, T.; Taguchi, H.; Shingu, K. Breakage of epidural catheters: a comparison of an arrow reinforced catheter and other nonreinforced catheters. *Anesthesia and analgesia*, **2001**, *92,* 246–248.

[6] Ugboma, S.; Au-Truong, X.; Kranzler, L.I.; Rifai, S.H.; Joseph, N.J.; Salem, M.R. The breaking of an intrathecally-placed epidural catheter during extraction. *Anesthesia and analgesia*, **2002**, *95,* 1087-9, table of contents.

[7] Lee, Y.-H.; Hwang, H.Y.; Sim, W.-S.; Yang, M.; Lee, C.J. Breakage of a thoracic epidural catheter during its removal -A case report-. *Korean journal of anesthesiology*, **2010**, *58,* 569–572.

[8] Pinciroli, R.; Fumagalli, R. The unexpected epidural: a case report. *BMC anesthesiology*, **2015**, *15,* 83.

[9] Kim, T.H.; Shin, J.J.; Lee, W.Y. Surgical treatment of a broken neuroplasty catheter in the epidural space: a case report. *Journal of medical case reports*, **2016**, *10,* 277.

[10] Hippalgaonkar, A.V.; Kudalkar, A.G.; Gaikwad, S.M.; Modak, S.; Gupta, H.B.; Tendolkar, B.A. Successful management of a broken epidural catheter!!! *Saudi journal of anaesthesia*, **2017**, *11,* 228–231.

[11] Powers, K.; Elmofty, D. Retained Intrathecal Catheter in a Patient With Baastrup Disease. *A&A practice*, **2020**, *14,* e01313.

[12] Patel, A.; Adsul, N.; Mahajan, S.; Chahal, R.S.; Kalra, K.L.; Acharya, S. Incidental unintentional breakage of epidural catheter in supralaminar area: A case report. *Surgical neurology international*, **2021**, *12,* 129.

[13] Taksande, K.; S., K.; Bhalerao, N.; Jadhav, J.; Wanjari, D.; Shatalwar, A. Case Report – Accidental Epidural Catheter Breakage and Its Management. *J. Pharm. Res. Int.*, **2021,** 1–5.

[14] Walia, S.; Pisal, T.; Kandari, A.; Jivrajani, P. Minimally Invasive Surgery to Remove a Broken and Retained Epidural Catheter Fragment. *Cureus*, **2022**, *14,* e25255.

[15] Sulhan, S.; Sadrameli, S.S.; Barber, S.; Holman, P.; Britz, G.; Huang, M. Fractured Lumbar Drain Catheter Retrieval Using an Endoscopic Transforaminal Approach to the Lumbar Spine. *Operative neurosurgery (Hagerstown, Md.)*, **2022**, *23,* e331-e334.

[16] Kong, W.; Du, Q.; Xin, Z.; Cao, G.; Liu, D.; Wei, Y.; Liao, W. Percutaneous fully endoscopic surgical management of the ruptured epidural catheter: Rescue of the novice anesthesiologist from his dilemma. *Frontiers in surgery*, **2022**, *9,* 915133.

[17] Gompels, B.; Rusby, T.; Slater, N. Fractured epidural catheter with retained fragment in the epidural space-a case study and proposed management algorithm. *BJA Open*, **2022**, *4,* 100095.

[18] Kumar, S.; Mahajan, S.; Kumar, V.; Gandhi, K.A. Broken epidural catheter: individualize your management. *Ain-Shams J Anesthesiol*, **2023**, *15,* 1–3.

[19] Motov, S.; Stienen, M.N. Extraction of a Torn Epidural Catheter in the Thoracolumbar Junction via Unilateral Biportal Endoscopy. *World neurosurgery*, **2025**, *193,* 1058.

[20] Alfadhel, A.; Turkistany, H.; Alkinani, A.A.; Kabbani, N.; Alabdullah, A.F. Broken Epidural Catheter After Vaginal Delivery. *Cureus*, **2024**, *16,* e55013.

[21] S, S.; Dussa, K.R.; Kokate, S.; Mukadam, M.; Zafar, S.; Choudhari, A. When The Unexpected Happens: The Intriguing Retrieval of a Broken Epidural Catheter. *Journal of orthopaedic case reports*, **2024**, *14,* 136–139.

[22] Tio, T.O.; Macmurdo, S.D.; McKenzie, R. Mishap with an epidural catheter. *Anesthesiology*, **1979**, *50,* 260–262.

[23] Moerman, N.; Porcelijn, T.; Deen, L. A broken epidural catheter. Case report. *Der Anaesthesist*, **1980**, *29,* 17–18.

[24] Staats, P.S.; Stinson, M.S.; Lee, R.R. Lumbar stenosis complicating retained epidural catheter tip. *Anesthesiology*, **1995**, *83,* 1115–1118.

[25] Blanchard, N.; Clabeau, J.J.; Ossart, M.; Dekens, J.; Legars, D.; Tchaoussoff, J. Radicular pain due to a retained fragment of epidural catheter. *Anesthesiology*, **1997**, *87,* 1567–1569.

[26] Collier, C. Epidural catheter breakage: a possible mechanism. *International journal of obstetric anesthesia*, **2000**, *9,* 87–93.
[27] Schummer, W.; Schummer, C. Another cause of epidural catheter breakage? *Anesthesia and analgesia*, **2002**, *94,* 233.
[28] Demiraran, Y.; Yucel, I.; Erdogmus, B. Subcutaneous effusion resulting from an epidural catheter fragment. *British journal of anaesthesia*, **2006**, *96,* 508–509.
[29] Pant, D.; Jain, P.; Kanthed, P.; Sood, J. Epidural Catheter Breakage: A Dilemma. *Indian Journal of Anaesthesia*, **2007**, *51,* 434–437.
[30] Rajendra, P.; Popham, P. Fracture of an epidural catheter inserted for labour analgesia. *Anaesthesia and intensive care*, **2008**, *36,* 245–248.
[31] Eap, C.; Frappart, M.; Litre, C.F.; Bourgeade, F.; Gomis, P.; Malinovsky, J.M.; Rousseaux, P. Minimally-invasive spinal surgery to remove a broken epidural catheter fragment. *International journal of obstetric anesthesia*, **2011**, *20,* 190–191.
[32] Drake, M. Broken epidural catheter. *Anaesthesia*, **2012**, *67,* 803–804.
[33] Abouhashem, S. Surgical removal of broken epidural catheter. *Saudi journal of anaesthesia*, **2013**, *7,* 96–98.
[34] Üşar, P.; Kar, A.A.; Çıtak, G.; Maral, J.; Canlı, Ş. Breakage of an Epidural Catheter Inserted for Labor Analgesia. *Turkish journal of anaesthesiology and reanimation*, **2015**, *43,* 282–284.
[35] Tarukado, K.; Oda, T.; Tono, O.; Suetsugu, H.; Doi, T. A Retained Epidural Catheter Fragment Treated by Surgery. *Asian Spine Journal*, **2015**, *9,* 461–464.
[36] Hösslin, T. von; Imboden, P.; Lüthi, A.; Rozanski, M.J.; Schnider, T.W.; Filipovic, M. Adverse events of postoperative thoracic epidural analgesia: A retrospective analysis of 7273 cases in a tertiary care teaching hospital. *European journal of anaesthesiology*, **2016**, *33,* 708–714.
[37] Ishikawa, Y.; Imagama, S.; Ito, Z.; Ando, K.; Gotoh, M.; Nishiwaki, K.; Nagao, Y.; Ishiguro, N. Delayed Onset of Subdural Hematoma following Epidural Catheter Breakage. *Global spine journal*, **2016**, *6,* e1-6.
[38] Reena; Vikram, A. Fracture of epidural catheter: A case report and review of literature. *Saudi journal of anaesthesia*, **2017**, *11,* 108–110.
[39] Siddappa, N.D.; Kim, J.-S.; Khandge, A.V. Full-Endoscopic Removal of Sheared Lumbar Epidural Catheter Fragment. *World neurosurgery*, **2020**, *137,* 421–424.
[40] Jiménez-Ponce, F.; Ramírez-Tapia, Y.; Ariñez-Barahona, E.; Nava-López, J.A.; Alla, S.N. Rare Image of Epidural Catheter Fracture in Lumbar Analgesia. *Case reports in anesthesiology*, **2023**, *2023,* 8880024.
